# Multimodality Imaging in Ischemic Cardiomyopathy

**DOI:** 10.1007/s12410-014-9285-x

**Published:** 2014-07-09

**Authors:** John O. Prior, Hoshang Farhad, Olivier Muller

**Affiliations:** 1 Department of Nuclear Medicine, Lausanne University Hospital, Rue du Bugnon 46, 1011 Lausanne, Switzerland; 2 Cardiovascular Division, Department of Medicine, Brigham and Women’s Hospital, Boston, MA USA; 3 Department of Cardiology, Lausanne University Hospital, Lausanne, Switzerland

**Keywords:** PET/CT, SPECT/CT, PET/MR, Myocardial perfusion, Ischemia, Cardiomyopathy

## Abstract

Cardiac multimodality (hybrid) imaging can be obtained from a variety of techniques, such as nuclear medicine with single photon emission computed tomography (SPECT) and positron emission tomography (PET), or radiology with multislice computed tomography (CT), magnetic resonance (MR) and echography. They are typically combined in a side-by-side or fusion mode in order to provide functional and morphological data to better characterise coronary artery disease, with more proven efficacy than when used separately. The gained information is then used to guide revascularisation procedures. We present an up-to-date comprehensive overview of multimodality imaging already in clinical use, as well as a combination of techniques with promising or developing applications.

## Introduction

**“Ischemic cardiomyopathy”** is defined as a weakness in the heart muscle due to inadequate oxygen delivery to the myocardium, with coronary artery disease (CAD) being the most common cause [1]. Thus, ischemic cardiomyopathy refers to a mix of ischemia and infarction, which is not currently supported in the AHA or ESC classification schemes [1]. The exact mechanism of ischemic cardiomyopathy is still under investigation and hibernating myocardium is presumed to be a complex adjustment to repetitive ischemia-reperfusion [2].

**Multimodality cardiovascular imaging** involves combination of at least two out the four existing cardiovascular imaging (nuclear cardiology with single photon emission computed tomography [SPECT] and positron emission tomography [PET], computed tomography coronary angiography [CTCA] and magnetic resonance imaging [MR]; for comprehensive reviews, see [3, 4]), the most frequent being PET/CT and SPECT/CT. There have been appropriate use criteria (AUC) published on the appropriateness of each technique for a given indication [5]. However, such criteria do not include hybrid-imaging technology yet. To adequately interpret such hybrid imaging, specialised cardiovascular imaging training programs have also been developed to include a 1- to 2-year multimodality component, and certification of physicians is now in discussion by professional bodies [6].

However, **hybrid (fusion) imaging** allows gaining important and complementary information and constitutes an opportunity for the future of cardiovascular imaging [7•]. Indeed, within the last decade, there has been a tremendous growth in cardiovascular imaging and there is a need to define imaging pathways for different clinical scenarios to identify cost-effective and diagnostically accurate algorithms [8]. In the developing “Image-Guided Therapy” paradigm, interventional cardiologists will need to gain information from non-invasive cardiovascular imagers to guide revascularisation efforts [9]. The integration of coronary anatomy and myocardial perfusion imaging has been recognised to offer improved diagnostic and prognostic information that could translate into improved patient care [10].

We will present an overview of the different combinations of hybrid imaging modalities to characterise ischemic cardiomyopathy, whether they are used frequently or are still in early development (Table 1).

**Table 1:** Cardiac imaging modalities that can be combined in hybrid fashion, with their publication frequency in the literature (●●● = frequently; ●● = occasional; ● = in development; ○ = experimental; Ø = no application described)

–	SPECT	PET	MR	CTP	ICA	CTCA
**SPECT** (single photon emission computed tomography)	–	Ø	SPECT/MR (○)	Ø	Ø	SPECT/CTCA (●●●)
**PET** (positron emission tomography)		–	PET/MR (●)	Ø	PET/ICA (○)	PET/CTCA (●●)
**MR** (magnetic resonance) gadolinium injection			–	Ø	Ø	MR/CTCA (●)
**CTP** (computed tomography perfusion) adenosine				–	Ø	CTP/CTCA* (●)
**ICA** (invasive coronary angiography)					–	ICA/CTCA (○)
**CTCA** (computed tomography coronary angiography)						–

*Not corresponding exactly to the definition of hybrid imaging as both perfusion and anatomical imaging originates from CT

## SPECT-Based Hybrid Techniques

### SPECT/CTCA

The addition of CT to SPECT allows attenuation correction and a better assessment of SPECT myocardial perfusion [11], although its use is not widespread and many centres continue to use simpler techniques such as prone imaging [12]. This combination of CT for attenuation correction is also not understood as hybrid imaging *per se*. In this overview, hybrid imaging is more thought of combining CTCA to SPECT in terms of diagnostic and prognostic value in ischemic cardiomyopathy. For a detailed, recent review on hybrid SPECT/CT imaging, we can recommend the reader to the latest work by Gaemperli et al. [13••]. These authors performed initial clinical testing [14] and sensed very early the potential for hybrid imaging in cardiology [15].

#### Added Value of Calcium Scoring

The use of a CT in combination with SPECT or PET allows assessing coronary artery calcium (CAC), which is a reproducible, inexpensive method to determine prognostic cardiovascular events rate [16, 17]. In combination with SPECT/CT, CAC was shown to unmask single or multiple vessel disease when highly elevated (CAC >1000) in patients with normal SPECT MPI [18]. In addition, it was shown to be a strong risk predictor of postoperative noncardiac surgery major cardiovascular events [19]. Even though limited evidence as to whether such imaging biomarkers lead to improved outcome, identification of coronary atherosclerosis by CAC scanning does lead to lifestyle changes and the intensification of medical therapies.

#### Added Value of Computed Tomography Coronary Angiography (CTCA)

**Fig. 1:**
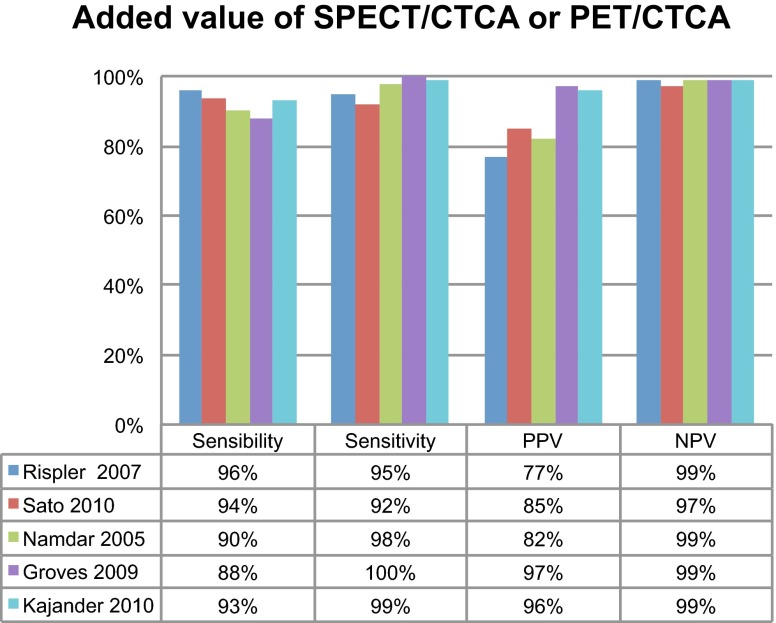
Diagnostic added value of SPECT/CTCA (Rispler et al. [20], Sato et al. 2010 [25]) and PET/CTCA (Namdar et al. [42], Groves et al. [43], and Kajander et al. [44]) as compared to invasive coronary angiography on a vessel-based analysis. Abbreviations: PPV = positive predictive likelihood; NPV = negative predictive likelihood

It is known that CTA alone performs poorly to predict myocardial ischemia with a low positive predictive value as compared with SPECT (29–58 %) [20–24]. However, the negative predictive value of a CTA examination is excellent (88–100 %) especially in a low risk population and practically excludes obstructive CAD and the need for further investigation. Thus, combining both SPECT with CTCA offers very good accuracy performances to detect hemodynamically significant coronary artery lesions [20, 25] (Fig. 1).

In an original study, Schaap et al. found that hybrid imaging with SPECT/CTCA led to similar treatment decision on revascularisation of coronary lesions by PCI or CABG in 107 patients with stable angina pectoris as SPECT followed by invasive coronary angiography [26]. In a similar study, Dong et al. found that SPECT/CTCA accurately detected functionally relevant coronary artery lesion as compared to ICA in 78 patients undergoing both examination within 1 month [27]. They concluded that SPECT/CTA could act as a potential gatekeeper for revascularisation. Of note, they observed that 37 % of the vascularised vessels were not associated with ischemia on myocardial perfusion imaging. Finally, Koukouradis and co-workers observed that SPECT/CTCA fusion imaging considerably altered the clinical decision for referral to further investigation [28]. Indeed, in 25 patients, 17 (68 %) were classified as having hemodynamically significant lesions based on SPECT imaging; when using SPECT/CTCA hybrid fusion, only six (24 %) were classified as having hemodynamically significant lesions.

#### Added Diagnostic Value of Hybrid Fusion Imaging as Compared to Side-by-side Analysis

**Fig. 2:**
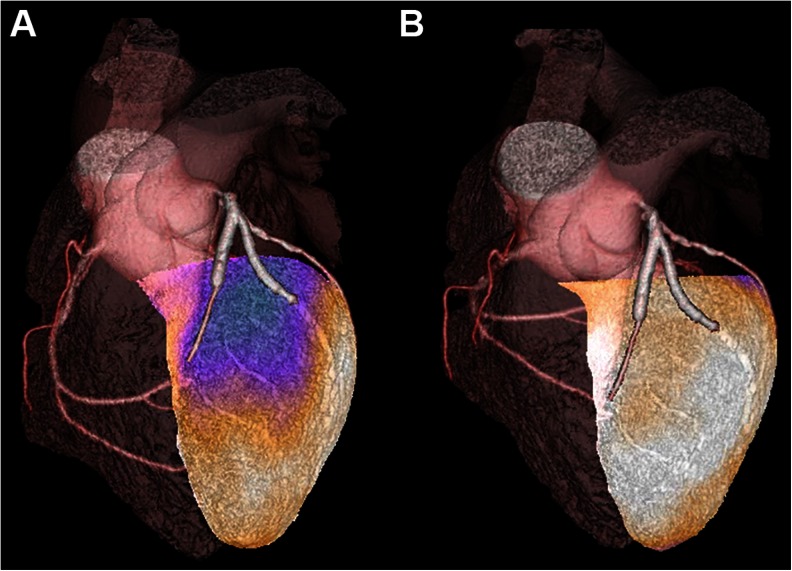
Example of hybrid SPECT/CTA under adenosine pharmacological stress in a 58-year male patient with two coronary stents (one implanted in the proximal left anterior descending [LAD] and one in the first diagonal branch) (**a**) A myocardial perfusion imaging was requested for investigating the start of angor similar as described before stent implantation. The CTCA did not show any intra-stent stenosis, but failed to visualize a small branch from the LAD which was recovered by the stent mesh and which was not visible anymore on CTCA, responsible for the symptomatic ischemia with a stress-induced perfusion defect (summed stress score = 10; summed rest score = 4; summed difference score = 6). There was unfortunately no possibility to revascularize this small branch and, consequently, maximal risk factor reduction and physical activity training were recommended. (**b**) After 9 months, myocardial perfusion imaging was repeated and fused with the same CTCA and regained normal perfusion at stress and rest, without ischemia and scar

Several studies have been comparing the added diagnostic value of hybrid fusion imaging as compared to separate side-by-side reading and found that it could confirm or exclude significant stenotic lesions in about 35 and 25 % [14], respectively, modify the initial diagnosis in 28 % of the cases [29] and increase diagnostic performances of finding the stenotic coronary artery in the LCX and RCA territories [30]. An example of SPECT/CTCA fusion is given in Fig. 2.

#### Added Prognostic Value of Hybrid Fusion Iimaging

**Fig. 3:**
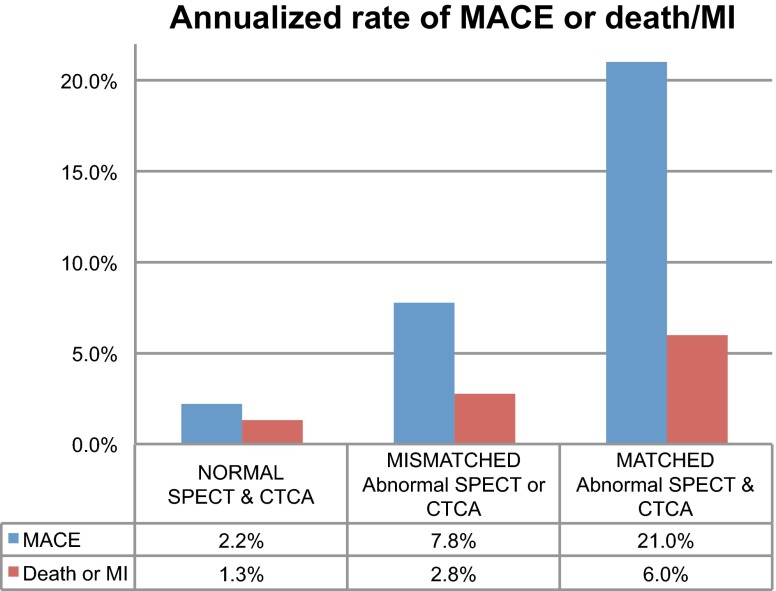
Annualized event rate of major acute coronary event (MACE: cardiac death, myocardial infarct, unstable angina requiring hospitalization and coronary revascularizations) and hard events (myocardial infarct [MI]/cardiac death) according to the outcome of the individual SPECT and CTCA studies stratified according to the yielded results and their agreement (matched vs. unmatched) (data from the study by Pazhenkottil et al. [31])

An important demonstration of the added prognostic value of hybrid imaging and its impact on patient management was performed by Pazhenkottil et al. [31]. In their follow-up study of 324 consecutive patients with known or suspected CAD referred for a 1-day stress/rest SPECT and a CTCA, they found valuable risk stratification information in hybrid imaging with an independent prediction of major acute coronary events (such as cardiac death, myocardial infarct, unstable angina requiring hospitalisation and coronary revascularisations). To this aim, they stratified patients according to the matching of both SPECT and CTCA into three groups: (1) reversible stress defect on SPECT and lesion on CTCA; (2) reversible stress defect on SPECT or a lesion on CTCA; and (3) normal SPECT and CTCA. They concluded that patients with matched hybrid defects were at higher risk than if findings were unmatched or normal (Fig. 3).

#### Perspective

The exact sequence in which to perform the SPECT and CTCA studies remains undetermined. However, a consensus seems to emerge in the literature with the personal likelihood of CAD being a fair option to orient this choice [32•]. In fact, patients with low pre-test likelihood of CAD could first undergo CTCA and stop further investigation if normal, thanks to the very high NPV of CTCA. If clearly positive or doubtful for a coronary lesion, the patient should undergo a SPECT (or PET) determination of stenosis significance on the myocardial blood flow. If the pre-test probability of CAD is higher or if the patient has known CAD, a SPECT would be performed first and if revascularisation either by PCI or CABG would be considered in the presence of multivessel disease, a CTCA would be performed.

### SPECT/MR

There might be interest in combining hybrid imaging from SPECT and MR, although no commercial SPECT/MR machine is sold today. Indeed, an interesting study found that in case of doubtful viability by MR, performing a gated-SPECT could detect viability or ischemia in up to 31 % of the segments with 50–75 % late-gadolinium enhancement [33]. These initial study questions will certainly also be addressed with existing PET/MR scanners.

## PET-Based Hybrid Techniques

### PET/CTCA

#### Diagnostic Superiority of PET over SPECT

PET has been at the origin of many studies on myocardial perfusion and myocardial viability [34]. It has many advantages over SPECT, including better spatial resolution and reliable attenuation correction, as well as a quantitative nature allowing obtaining absolute myocardial blood flow, and lower radiation burden [35]. Recent PET scanners are most often coupled with CT and are therefore hybrid by design (PET/CT). However, most of the time, the CT is only used for attenuation correction. The incremental value for predicting future cardiovascular events and cardiac death due to myocardial blood flow quantitation has also been demonstrated over SPECT in several studies [36–40]. The scientific basis for clinical cardiac PET and its use to guide management and revascularisation has been recently reviewed [41], and is awaiting confirmation in randomised controlled multicentre trials.

As with SPECT/CTCA, the addition of CTCA to PET offers prognostic information, which is not available with PET/CT alone in the presence of obstructive CAD [42–44] (Fig. 1) and even nonobstructive CAD [45–47], despite an incremental prognostic value of CTCA over CCS [48].

A study by Danad et al. evaluated the impact of O-15-water PET/CTCA imaging on ICA referral and revascularisation in 375 patients [49]. The authors found that in the presence of equivocal (21 % of the patients) or abnormal CTCA (30 % of the patients), MPI could guide the decision for ICA referral and revascularisation.

In a recent head-to-head comparison study, Thomassen et al. found that CTCA and O-15-water PET had better diagnostic accuracy than CTCA or PET alone in 44 outpatients scheduled for ICA [50]. Furthermore, the presence of suboptimal CTA PPV (71 % [95%CI 53–85 %] on a per-patient basis and 53 % [39–66 %] on a per-vessel basis) suggested that hybrid PET/CTCA could complement the assessment of coronary stenosis diagnosed by CTA (PET/CTCA PPV: 100 % [84–100 %] and 85 % [73–97 %], respectively).

### PET/MR

The utility of PET/MR has been speculated before integrated hybrid prototypes were available [51]. Potential utility in comparison to stand-alone scanners, is for instance MR-based attenuation, motion and partial volume correction, improved prognostic stratification of LV function and metabolism in heart failure patients, as well as detailed risk assessment infarction and viability [51, 52•]. Today, there have been only a limited number of combined studies of nuclear perfusion imaging with MR and more studies are needed to fully understand the use of multimodality PET/MR techniques in ischemic cardiomyopathy. Current reviews on the potential of PET/MR have been published recently [52•, 53, 54•, 55, 56], but the true value seems to be less clear than in the domain of oncology [53], without any clear obvious indications or “killer” applications identified yet [52•]. Stress perfusion MRI has a good sensitivity and specificity for detecting ≥50 % stenosis in recent metaanalyses [57, 58]. Unlike PET/CT, spatial and temporal correlations are possible with PET/MR. Also, MR coronary angiography is currently developing better spatial resolution and measurement of intracoronary blood flow [59], which will eventually lead to clinical applications. Furthermore, left ventricular ejection fraction, end-diastolic and end-systolic volumes have prognostic implication and PET-derived regional myocardial glucose consumption gives complementary information. Finally, MR might be able to continuously monitor patient movement and eventually obtain better PET quantitation [52•].

Image interpretation will need a new generation of well-trained specialists both in PET and MR fields, which represent an opportunity, but also a challenge for certification [6, 56].

In an initial study with fully integrated PET/MR, Nensa et al. demonstrated the feasibility of this approach in 20 consecutive patients with myocardial infarct using F-18-fluorodeoxyglucose (F-18-FDG), with the conclusion that it may add complementary information in patients with ischemic heart disease [60].

Limitations of cardiac MR exist, such as claustrophobia (6–15 % [61–63]), which is more frequent due to smaller and longer PET/MR bore [56], impossibility to scan patients with non-MR-compatible implants (pacemaker, defibrillators, metallic clips) or inject contrast in patients with decreased renal function (creatinine clearance rate <30 mL/min/1.73 m^2^).

#### Added Value for Prognosis

In a study in 151 consecutive patients with suspected CAD, Chen et al. found that stress MR and CTCA were highly concordant (92 % agreement) and negative test results conferred excellent prognosis of event-free survival (MR 97 %, CTA 99 %) with an average follow-up of 450 ± 115 days [64].

#### Cost-Effectiveness

So far, combined PET/MR appears to be promising for evaluating cardiovascular diseases, but there has not been any study to determine whether such highly complex and costly technology is cost-effective, especially taking into account that the initial investment in a PET/MR scanner is about twice the cost of a PET/CT scanner.

### Right Heart Hybrid Imaging

**Fig. 4:**
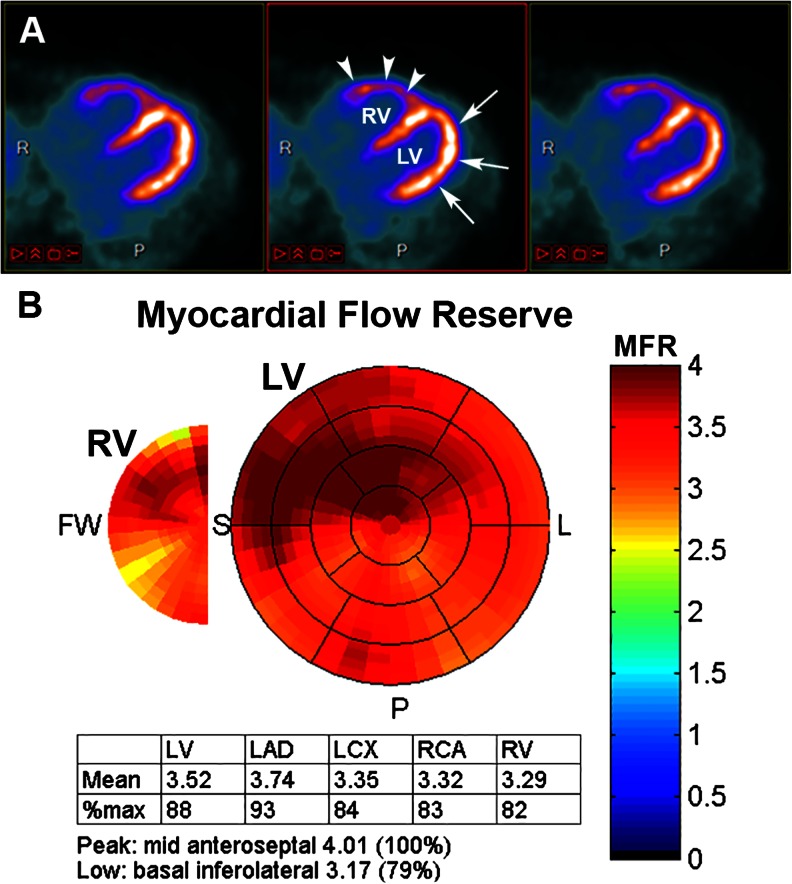
(**a**) Three transaxial slices of cardiac Rb-82 myocardial perfusion imaging in a 55-year women with pulmonary arterial hypertension showing the left ventricle (LV, arrow) and the hypertrophied right ventricle (RV, arrowhead), usually only faintly visible. (**b**) Corresponding polar map quantification of myocardial flow reserve of the left and right ventricle, with corresponding MFR in each territory and the right ventricle

Right ventricle (RV) is an important determinant of outcome in different cardiovascular and pulmonary diseases [65]. The use of multimodality imaging with MR and echography is current for assessing structure and function, while due to technical stagnation, nuclear imaging for the RV has become obsolete nowadays [65]. Unless in the pathologic state of hypertrophied RV, such as in pulmonary arterial hypertension, RV is difficult to visualise and might be better seen with PET than SPECT [65]. It becomes possible to quantify myocardial blood flow in the right ventricle (Fig. 4) and the feasibility of hybrid imaging with PET/CTA for the RV remains to be demonstrated.

### PET/ICA

**Fig. 5:**
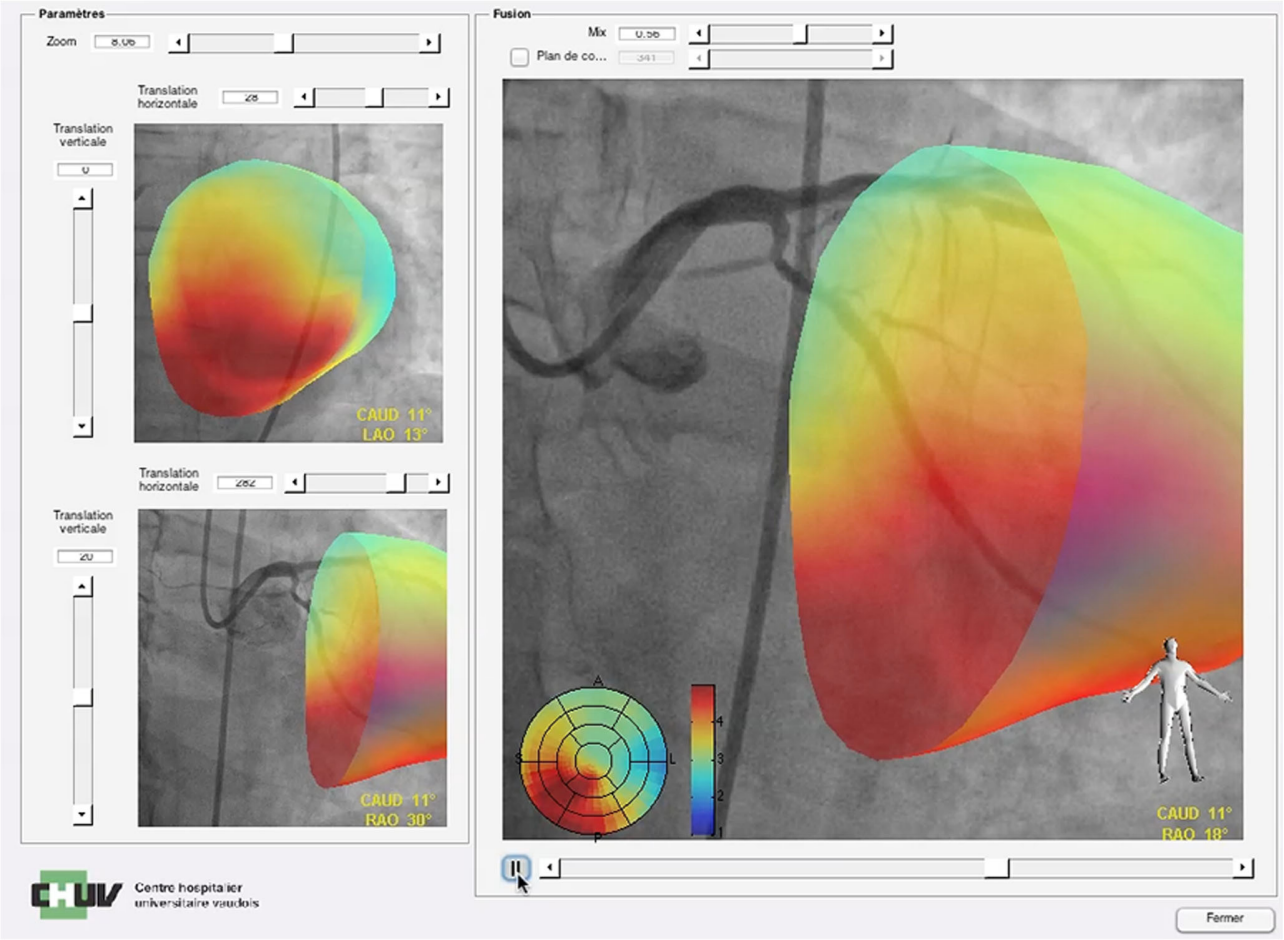
Example of PET/ICA hybrid imaging with our user interface allowing live fusion of PET myocardial blood flow information (in colour) onto angiographic projection of rotational X-ray angiograms (in black and white) in the catheterization laboratory

A tool that enables going directly from PET imaging into the catheterisation laboratory without additional radiation burden would be desirable. We started to develop such a tool able to reproject the PET myocardial blood flow image onto angiographic projection of rotational X-ray angiograms (Fig. 5). The potential utility of such an approach needs still to be evaluated into a pilot study, but would certainly be attractive for the interventional cardiologist, allowing him to rapidly visualise the coronary branch responsible for the limited myocardial perfusion, without performing any supplementary CTCA or injecting additional contrast material, with only a radiation burden.

## Other Non-Nuclear Hybrid Techniques

### Hybrid Imaging with Echography

An interesting approach would be the possibility to obtain three-dimensional information from myocardial contrast echography to image myocardial perfusion and to perform fusion imaging with functional SPECT/PET information [66] or with morphological CT/MR information [67]. The image fusion of real-time 3-D echography and ICA for guidance during catheterisation procedures has been demonstrated and it was found clinically useful not only for mitral clip implantation but also for AF ablation and TAVI aortic valve replacement [68]. So far, such hybrid echographic combinations have not emerged into clinical applications for ischemic cardiomyopathy.

### Intracoronary Optical Coherence Tomography (OCT)

Intracoronary optical coherence tomography (OCT) is a catheter-based invasive imaging system that provides accurate measurements of intraluminal architecture and plaque characterisation [69]. It can be fused with hybrid imaging such as intravascular ultrasound (IVUS) or CT [70], but it is only emerging as a hybrid imaging modality, and awaits further development.

### Myocardial CT Perfusion (CTP)/CTCA

Although dynamic CT perfusion (CTP) performed under adenosine stress and CTCA are not exactly “hybrid” as they are both from the same CT scanner, they represent a fast and interesting way of probing the myocardial perfusion and the coronary arteries.

#### Added Diagnostic Value

CTP is still in development [71] and comparisons performed with coronary blood flow and fractional flow ratio (FFR) are good in animals [72]. Furthermore, diagnostic performance of detecting functionally significant coronary lesions was improved by a dynamic stress CTP-derived index of myocardial blood flow over CTCA in 80 symptomatic stable patients with intermediate pre-test CAD likelihood referred to ICA indicating potential for use in patient management [73], although an accompanying editorial outlined several of the study limitations (e.g., lack of rest study hindering distinguishing myocardial scar from ischemia) and outlined the need for a larger prospective multicentre trial in unselected patients [74].

Two recent studies have shown good results as compared to nuclear techniques. In a first study, CTP and CTCA could correctly identify functionally significant ≥50 % stenosis on SPECT in a large 16-centre study in 381 patients [75]. In the second study, CT myocardial adenosine stress and rest myocardial perfusion was compared to O-15-water PET myocardial blood flow using a CT-derived method to compute coronary flow reserve (CFR) with a low-dose 320-slice CT in 32 subjects (12 pilot volunteers, 13 validation volunteers and seven CAD patients) [76]. There was a strong correlation between PET-derived MBF and CTP-derived MBF (*r* = 0.95, *P* < 0.0001) and lower CFR in the CAD group than in the validation group of volunteers (2.3 ± 0.8 vs. 5.2 ± 1.8, *P* = 0.0011). This established the feasibility of measuring MBF and CFR by CTP, thus opening new potential applications of hybrid imaging.

### CTCA/ICA

Preprocedural CTCA can be of interest in preparing percutaneous intervention (PCI) for chronic total occlusion, which is present in about 20 % of patient with known or suspected CAD. In their study, Rolf et al. found that three-dimensional rendering of the CTCA displayed in the catheter lab during the PCI helped to raise the success rate in complex lesions, with success rate of 88 % (22/25 patients) as compared to 63 % (16/25 patients matched) (p = 0.03) [77].

## Future Perspectives

### Radiation Burden and Other Risks

Radiation burden was found initially to be one of the limitations of hybrid imaging with nuclear and CT combined techniques. However, it underwent substantial reduction over the last decade by at least an order of magnitude and will undoubtedly still benefit of further reduction in the future. Nevertheless, imaging is coupled with risks that are often not limited to ionizing radiations only; a good review on the risk of imaging coronary artery disease with ionizing radiation as well as MR and echography has been recently published, including risks from stressors, contrast agents, and invasiveness, among others [78••]. The review concluded that coronary disease imaging, including ICA, is bound to small, but detectable acute and long-term risks that are in the same range of common daily-life activities (e.g. fatal pedestrian accident risk or about 15 % of fatal motor vehicle accident risk) and only a fraction of common therapeutic medical intervention (10–20 % lifetime risk of fatal aspirin bleeding) and only a minor fraction (2 %) of the risk of lifetime fatal cardiac event [78••].

### Molecular Hybrid Imaging

**Fig. 6:**
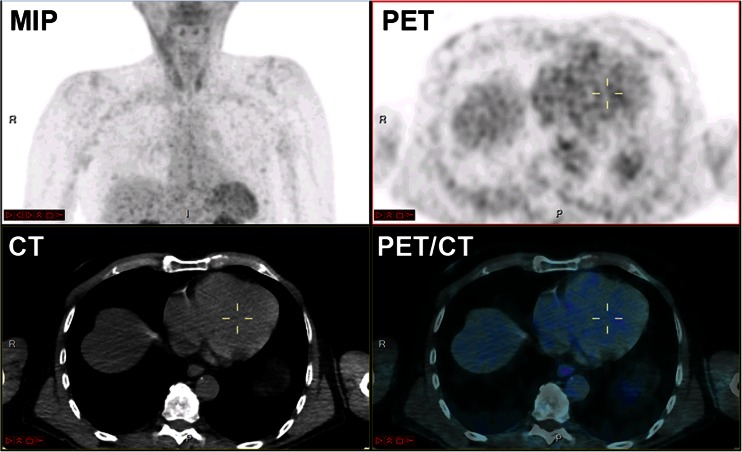
Example of Ga-68-NODAGA-RGD for imaging neoangiogenesis in a 72-year male patient with history of cardiac infarct 7 years prior to PET/CT imaging. Selected transaxial slice through the heart at 70-min post radiopharmaceutical injection showing no abnormal uptake in the old myocardial infarct indicating that no active angiogenesis is taking place

With the advent of SPECT or PET showing molecular imaging targets involved in the development of vulnerable plaque, the process of myocardial infarct or the development of heart failure, such as neoangiogenesis (Fig. 6) or apoptosis, the need for precise localisation of the observed abnormalities with CT or MR would be increased [52•, 79, 80].

### Cost-Effectiveness

The Study of Myocardial Perfusion and Coronary Anatomy Imaging Roles in Coronary Artery Disease (SPARC) register collected clinical and economical data on 1703 patients with suspected CAD undergoing CTCA, PET and SPECT in a standardised fashion with a 2-year clinical follow-up [81]. This study found that 2-year costs were lower after using SPECT rather than CTA or PET [82]. They concluded that SPECT was more economically attractive as compared to PET, whereas CTA was associated with higher costs and no significant difference in mortality as compared to SPECT. In an accompanying editorial, Villines and Min remarked that mean costs were higher for CTA and PET as compared to SPECT, stemming from higher rates of ICA and revascularisation [83]. They further question whether this underscores superior diagnostic performances of CTA and PET as compared to SPECT or just higher rates of revascularisations. They also salute the step taken from the SPARC authors in finding new metrics, which reveals more complexity in the study results analysis. Yet study results are heterogeneous and many centres did not offer the three modalities rendering head-to-head comparison impossible. They also note the considerable technological progress made from the inception of SPARC in 2006 in terms of CTA (iterative reconstructions, dual-energy CT, etc.) that outpaced the study. Despites many limitations, this study should be commended for asking the right questions (which test, when?) and by leading the field beyond traditional diagnostic and prognostic performances [83].

## Conclusion

The utility of hybrid nuclear imaging with PET/CT is well recognised in oncology, but in cardiology, its utility is less obvious. Evidence for a clinical utility has accumulated in the last decade, and a body of proof now exists. Nonetheless, due to higher costs, low availability and additional radiation exposure, there are no clear recommendations that have been implemented into guidelines yet, except a position statement on the use of hybrid imaging in patients with known or suspected CAD [32•].

Hybrid imaging has a role to play in characterising ischemic cardiomyopathy as a gatekeeper to the more invasive coronary angiography in the setting of more personalised medicine. On-going (EVINCI, SPARC) and future large, multicentric studies will have to particularly address the cost efficiency and accuracy, as well as the complementarity of the different hybrid imaging pathways to help clinicians optimising patient care.
